# The comparative performance of DBS artefact rejection methods for MEG recordings

**DOI:** 10.1016/j.neuroimage.2020.117057

**Published:** 2020-10-01

**Authors:** Ahmet Levent Kandemir, Vladimir Litvak, Esther Florin

**Affiliations:** a Institute of Clinical Neuroscience and Medical Psychology, Medical Faculty, Heinrich-Heine University Düsseldorf, Germany; b Wellcome Centre for Human Neuroimaging, 12 Queen Square, London, UK

**Keywords:** MEG, DBS artefacts, Filtering, Hampel filter, tSSS, S3P, ICA-MI

## Abstract

Deep brain stimulation (DBS) can be a very efficient treatment option for movement disorders and psychiatric diseases. To better understand DBS mechanisms, brain activity can be recorded using magnetoencephalography (MEG) with the stimulator turned on. However, DBS produces large artefacts compromising MEG data quality due to both the applied current and the movement of wires connecting the stimulator with the electrode. To filter out these artefacts, several methods to suppress the DBS artefact have been proposed in the literature. A comparative study evaluating each method’s effectiveness, however, is missing so far.

In this study, we evaluate the performance of four artefact rejection methods on MEG data from phantom recordings with DBS acquired with an Elekta Neuromag and a CTF system: (i) Hampel-filter, (ii) spectral signal space projection (S3P), (iii) independent component analysis with mutual information (ICA-MI), and (iv) temporal signal space separation (tSSS). In the sensor space, the largest increase in signal-to-noise (SNR) ratio was achieved by ICA-MI, while the best correspondence in terms of source activations was obtained by tSSS. LCMV beamforming alone was not sufficient to suppress the DBS-induced artefacts.

## Highlights

• Phantom MEG measurement with Elekta Neuromag and CTF MEG system with DBS.
• Systematic comparison of cleaning algorithms to remove DBS artefact from MEG data.
• Sensor level ICA-MI yielded the best results.
• Source level: tSSS provided the best correspondence to recording without DBS.

## 1 Introduction

Deep Brain Stimulation (DBS) is an invasive treatment option for neurological and psychiatric disorders (Wichmann and DeLong, 2006), which can improve the patient’s quality of life substantially. Despite its clinically established use for movement disorders in particular (Lozano and Lipsman, 2013), its underlying mechanisms still remain elusive (Harmsen et al., 2018; Udupa and Chen, 2015). To better understand the mechanisms of DBS, magnetoencephalography (MEG) is potentially a powerful tool due to its high temporal and relatively good spatial resolution (Harmsen et al., 2018). MEG is a noninvasive and passive technique which does not pose any risk to subjects but is highly susceptible to interference of magnetic fields caused by DBS. Employing MEG in combination with DBS therefore requires suitable cleaning algorithms to remove DBS artefacts.

There are two types of artefact with different characteristics that need to be removed when recording patients with a DBS system implanted in the MEG. The first type of artefact produced by DBS systems is directly due to the stimulation with electric pulses. Electrical stimulation used for clinically-effective DBS consists of narrow pulses (60 μs - 200 μs) delivered at frequencies larger than 70 ​Hz to targeted brain areas (Lio et al., 2018). DBS pulses are square waves and therefore have infinite odd harmonics of the fundamental frequency (Oppenheim et al., 1997). Consequently, to completely digitize the stimulation pulses requires an infinite sampling rate. In practice, actual MEG systems sample at a few kHz at most, which - in combination with the aforementioned DBS characteristics - results in missed or under-sampled DBS pulses (Lio et al., 2018). Inconsistently sampled DBS pulses in turn give rise to peaks all over the frequency spectrum (Jech et al., 2006; Sun et al., 2014), which can also not be taken care of by the hardware low-pass filter.

The second type of artefacts is induced by movement of the hardware of the DBS system, i.e. the extension wires, connectors placed on the skull, and the DBS generator itself. Ferromagnetic percutaneous wires and connectors, which move due to arterial pulsation, can create strong artefacts in multiple overlying channels (Litvak et al., 2010). Accordingly, MEG recordings of DBS-implanted patients have the strongest artefacts on channels closest to the wires and connector (Airaksinen et al., 2011). Consequently, the combination of these different types of DBS-related artefacts is complex and they cannot be easily eliminated using conventional filters.

Methods to remove the DBS-related artefact exploit either spatial (Airaksinen et al., 2012; Boring et al., 2019; Hirschmann et al., 2013; Taulu and Simola, 2006) or spectral information (Allen et al., 2010; Dagar et al., 2018; Lio et al., 2018; Ramírez et al., 2011) of the recording. More recently, a new method based on temporal decomposition of the MEG sensor data produced promising results (ICA-MI (Abbasi et al., 2018, 2016)). However, a systematic evaluation and comparison of the respective methods’ ability to remove the DBS artefact from the data is still lacking.

In the present study we employ MEG phantom recordings with DBS to fill this gap. This approach yields the unique opportunity to investigate the effects of DBS artefacts on MEG data by comparing results to a ground truth. We recorded MEG data once with an Elekta Neuromag system and once with a CTF system from phantoms with physiological activity mirrored by a dipolar source oscillating at 12 ​Hz and a DBS stimulation setup that used movement mimicking the pulsation. An ideal cleaning algorithm would remove all DBS-related artefacts while preserving the dipole activity. With this goal in mind, we included four artefact rejection methods which are applied at the sensor level: (i) Hampel-filter (Allen, 2009), (ii) spectral signal space projection (S3P) (Ramírez et al., 2011), (iii) independent component analysis with mutual information (ICA-MI) (Abbasi et al., 2016), and (iv) temporal signal space separation (tSSS) (Taulu and Simola, 2006). These methods were systematically evaluated at both sensor and source level. We restrict our analysis to sensor level-based DBS artefact rejection methods as the goal is to obtain artefact-free sensor level data that is amenable to a wide range of subsequent analysis techniques. For this reason, our analysis does not include approaches that can only remove artefacts at the predefined DBS stimulation frequency and its harmonics but not e.g. movement artefacts.

## 2 Materials and methods

### 2.1 Data acquisition

To evaluate the effectiveness of different artefact rejection methods, we recorded from DBS phantoms with two major MEG systems: a 275-Channel VSM/CTF and a 306 Channel Elekta Neuromag (Vectorview) MEG system. All data are available at https://openneuro.org/datasets/ds002885.

#### 2.1.1 CTF recordings

For the present study, 273 of the 275 channels could be recorded. The experimental setup was similar to the one described in Oswal et al. (2016): Within the phantom, a dipole and a DBS electrode were placed. Additionally, a reference electrode was inside the phantom to record the electrical activity. An inflatable, non-magnetic tube was placed under the phantom to mimic arterial pulsations. An in-house pneumatic controller, which was placed outside the shielded room, inflated the tube every 2.5 ​s for ~40 ​ms. The deflation lasted between 500 and 1000 ​ms, causing the phantom to move ~3 ​mm vertically. We used a Medtronic (Medtronic Neurological Division, Minneapolis, MN) external stimulator (type 3628) to administer DBS through a Medtronic DBS electrode (model 3389). Abbott DBS wires (Abbott Laboratories, Abbott Park, Illinois) were taped to the phantom to make the measurements as realistic as possible. DBS stimulation parameters mimicked clinically relevant settings for Parkinson’s patients (Groiss et al., 2009): 130 ​Hz in bipolar mode with 140 μs pulse width and 3.1 ​V amplitude. The recordings were made with an electric dipole oscillating at 12 ​Hz. The power of the dipole was adjusted to achieve a ratio of DBS peak power to dipole peak power that is similar to the ratio of DBS peak to alpha peak power in a patient recording with similar DBS stimulation settings (~1.75 in logarithmic power scale). The sampling rate for the MEG data was 19.2 ​kHz in order to reduce aliasing effects.

To identify the noise patterns introduced by DBS, the attached wires, and the movement of the phantom, we performed separate measurements of 4 ​min length. The first measurement (termed DMW) was with the dipole turned on, tube movement and the wires attached. This type of recording mimics a patient recording with the DBS stimulator implanted, but not yet turned on. The second combination, termed DSMW (Dipole, Stimulation, Movement, Wires), mimics a patient recording with DBS turned on. It included the dipole activity and all of the described noise sources. To evaluate the cleaning effectiveness of the different algorithms, we also recorded the phantom with only the dipole turned on, while all artefact sources were turned off. This recording serves as our reference recording, termed Reference in the following. In an ideal scenario, the noise-contaminated recording should be the same after cleaning as the reference recording. Finally, in order to capture sensor and environmental noise, we recorded empty room data.

#### 2.1.2 Elekta Neuromag recordings

For the Elekta Neuromag recordings, we utilized a pneumatic movement mechanism which applied a repeated, regular motion to the DBS phantom as in CTF recordings. The movement mechanism basically consisted of a nylon-covered box which acted as a diaphragm when air was pumped inside. The phantom was placed on top of the box and air was pumped inside the box every 2.5 ​s for ~40 ​ms. Total displacement of the phantom was ~3 ​mm. We used a magnetic dipole oscillating at 12 ​Hz to mimic the brain activity and an Abbot St. Jude’s DBS system (Abbott Laboratories, Abbott Park, Illinois) was employed to administer DBS. In order to obtain a similar dipole power/DBS power ratio as for the CTF recordings, we set the parameters of the DBS system to 5 ​mA current and 60 μs pulse length. Although DBS frequency was set to 130 ​Hz, frequency analysis revealed the peak at 127.75 ​Hz. We performed three measurements of 2 ​min each: DSMW condition, reference, and empty room. The sampling rate for the MEG data was 3000 ​Hz.

### 2.2 Signal processing

All analyses were done in MATLAB (R2017a, The Mathworks Inc., Natick, MA) using Brainstorm (Tadel et al., 2011). Fieldtrip (Oostenveld et al., 2011) and SPM12 (Litvak et al., 2011) were also utilized as auxiliary toolboxes for ICA-MI and tSSS implementations, respectively. The customized Matlab code is available at https://gitlab.com/lkandemir/dbs-artefact-rejection. In both MEG systems, prior to any analysis, we used device-specific noise suppression techniques. For CTF data, the 3rd order gradient compensation was applied to reduce magnetic disturbances originating from outside the MEG helmet. For Elekta Neuromag data, we applied the system’s predefined SSPs to suppress magnetic interference originating from outside the MEG chamber. In both systems, notch filter with default settings of Brainstorm was used to attenuate power line noise at 50 ​Hz and its harmonics (up to 300 ​Hz in CTF and 500 Hz in Elekta Neuromag recordings). As DBS artefact removal was the main aim of the present study, band-pass or low-pass filtering was not considered. To reduce the computational cost for the CTF data, the data were down-sampled from 19.2 ​kHz to a sampling frequency of 2400 ​Hz. This is also a commonly used sampling rate in MEG studies (Florin and Baillet, 2015; Hirschmann et al., 2020; Oswal et al., 2016). Down-sampling was not necessary for Elekta Neuromag data, because it was sampled with only 3000 ​Hz ​at acquisition. All recordings were visually inspected to detect and reject artefactual segments caused by SQUID jumps or transient effects of the notch filter. For comparability, we removed the same time segments for all recordings within each data set. This resulted in a total data length of 216 out of 240 ​s for CTF and 117 out of 120 ​s for Elekta Neuromag recordings. Similarly, the frequency spectra were visually inspected to detect channels which were flat or showed substantially higher or lower noise levels than the average channels. None of the channels of the CTF recordings had to be excluded. However, 13 channels had to be excluded from Elekta Neuromag recordings.

### 2.3 DBS artefact rejection methods

#### 2.3.1 Hampel filter

The Hampel filter aims to reject narrow frequency peaks caused by DBS (Allen, 2009). Narrow frequency peaks are considered outliers from the frequency domain perspective. Sensor data was transformed to frequency domain using the Fast Fourier Transform (FFT). Then a so-called Hampel identifier was used on sliding windows of frequency-domain sensor data to detect such outliers both in the real and imaginary part of the frequency spectrum. Detected outliers are replaced with the median of the current window. The spectra filtered this way are transformed back to the time domain via inverse FFT (IFFT). FFT and IFFT operations were applied without windowing with default parameters in MATLAB. The sensitivity of the Hampel identifier is fine-tuned by changing the threshold parameter *C,* which determines the number of standard deviations the spectrum needs to be above the median of the spectrum within a frequency window to be classified as an outlier. Higher values indicate a lower sensitivity.

The size of the sliding window is chosen based on the width of the artefact, which was 0.2 ​Hz for the DBS peak. In order to achieve sufficient artefact suppression, at least twice the size of the artefact should be used. Overall, a larger window size increases the computational costs. Thus, we evaluated window sizes of 0.5 ​Hz, 6 ​Hz, and 10 ​Hz respectively (see Supplementary Fig. 1) for the CTF recordings. 6 and 10 ​Hz windows led to better results than a 0.5 ​Hz window, but results did not differ between the 6 and 10 ​Hz window. Therefore, a 6 ​Hz window was used for the further analysis. Previous EEG studies suggest a threshold parameter *C* ~5 (Allen, 2009; Davies and Gather, 1993), while in an EEG study the DBS-related artefacts were removed with *C* ​= ​6 (Allen et al., 2010). In the present study, we varied the threshold parameter *C* between 1 and 8 to evaluate the outcome depending on *C*.

#### 2.3.2 Spectral signal space projection algorithm (S3P)

S3P spatially projects the traces of the artefact out of the spectrum (Ramírez et al., 2011). In order to achieve this, cross-spectrum-density (CSD) matrices of sensors at each frequency are obtained from time-frequency (TF) decomposed MEG recordings. The choice of window length for S3P should depend on the frequency width of the artefact. If a frequency resolution lower than the actual width of the artefact is chosen, unnecessary suppression in adjacent frequencies would be observed. This is due to the fact that the CSD in each frequency bin is evaluated individually. DBS artefacts are usually narrow peaks of 0.2 ​Hz width, suggesting that the choice of a 0.25 ​Hz frequency resolution adopted in the present study is adequate. Therefore, also following the original paper of Ramírez et al. (2011), we Fourier transformed 4 ​s windowed sensor data with 50% overlap using a Kaiser window.

For each CSD matrix, the eigenvectors were obtained. In a second step, at every frequency f, frequency-specific spatial projectors P(f) were calculated based on a predefined number k(f) ​> ​0 of eigenvectors:

$$P\left(f\right)=I-U_{k\left(f\right)}{U_{k\left(f\right)}}^{H}.$$

$U_{k\left(f\right)}$ is a matrix containing the first k frequency-specific eigenvectors that span the noise subspace at frequency f. I denotes the identity matrix and H the Hermitian transpose.

These spatial projectors were then multiplied with TF data at each frequency. Finally, the cleaned data are obtained by taking the inverse Fourier transform of this TF data. Importantly, the spatial projector P(f) should be calculated from data segments in which the artefact to be removed is strong. This can be problematic for DBS artefact rejection because signals are always mixed with intrinsic brain activity. In the original paper it was proposed that frequency bands containing artefacts should be defined and the S3P algorithm should be applied only on those bands. However, this can be impractical because DBS artefacts are usually evident across the entire frequency spectrum, including physiologically relevant frequencies (Lio et al., 2018). In order to give the algorithm a fair chance of removing all artefacts in our controlled setting we applied S3P once to the DBS frequencies only and once to the complete spectrum (denoted wide band (WB) subsequently). We varied the number of eigenvectors between 1 and 9 to obtain the best-performing algorithm setting. This is also the range evaluated within the original paper (Ramírez et al., 2011).

#### 2.3.3 ICA-MI

ICA-MI exploits independent component analysis (ICA; Comon (1994)) in order to decompose the MEG recording into its components (Abbasi et al., 2016). The components of the artefact are later distinguished from the intrinsic activity by calculating mutual information (MI; Hudson, 2006) between the independent components and a simultaneous reference signal of the DBS. In actual patient measurements, such a reference signal is acquired using surface electrodes placed on the implanted stimulator or the extension wires and should mainly contain information about artefacts like the stimulation signal, movement, and cardio-ballistic impulses. In order to obtain the reference signal of the DBS artefact in CTF recordings, we recorded from the reference electrode inside the phantom. In case of Elekta Neuromag recordings, we used the MEG channel showing the largest peak at the DBS frequency as a reference signal (MEG 2413). We then applied a notch filter at 12 ​Hz to the reference signal, yielding an actual reference signal without dipole activity. For the removal of potential artefactual ICs a threshold is selected based on the mutual information (MI) between ICs and the reference signal. The idea is that ICs sharing a lot of information with the reference signal are likely representing the artefact in the MEG recording. Non-rejected ICs are transferred back to the sensor level to obtain artefact-free recordings. The crucial part of ICA-MI is to select an appropriate threshold for IC removal. The literature proposes to select the threshold based on visual inspection because no objective criterion has yet been developed. However, this may not be operational as it requires a lot of user experience. In order to evaluate the effect of the threshold, we evaluated a range of 5%–40%, covering also the range reported in previous studies (Abbasi et al., 2018, 2016).

ICA decomposition was achieved using the extended Infomax algorithm (Bell and Sejnowski, 1995). For the recordings of both MEG systems, the initial full ICA did not converge due to rank deficiency caused by the low number of signal sources compared to the high number of MEG channels. Therefore, before ICA, the data was reduced to ~75% of its first principal components. PCA reduction yielded 200 principal components for CTF recordings (99.9% of data variance explained) and 216 principal components (71 magnetometers - 99.9% data variance explained, 145 gradiometers – 98.3% data variance explained) for Elekta Neuromag recordings.

#### 2.3.4 Temporal signal space separation (tSSS)

tSSS (Taulu and Simola, 2006) is an extension of signal space separation (SSS; Taulu and Kajola, 2005) whereby the data are segmented temporally and each segment is analyzed separately. The length of the data segments has to be specified by the user. For each segment the sensor data are separated into three parts using the geometrical information of the sensor array and Maxwell equations: an internal part corresponding to source locations inside the sensor array, an external part corresponding to sources outside the MEG helmet, and an intermediate part lying in between. The intermediate part should be free of magnetic sources. But that would not be the case if there is a very strong source whose activity leaks either from the internal or the external part to the intermediate part (Taulu and Hari, 2009). Such a source would be considered noise. tSSS now assumes that intrinsic brain activity is uncorrelated with artefacts. Therefore, any temporal correlation between the intermediate and internal parts needs to occur as a result of noise mixture and is detectable by a subspace intersection method (Golub and Van Loan, 1996). Signals showing higher correlation values between intermediate and internal parts than a pre-defined correlation limit (CL) are projected out of the internal parts.

tSSS was first developed for Elekta Neuromag MEG systems and delivered to end users embedded in a noise suppression software named MaxFilter. For CTF system, however, a MATLAB implementation was also adapted by Samu Taulu and made available by MEGIN Oy (Helsinki, Finland) as a toolbox for SPM12. In order to process CTF data, we used the MATLAB implementation with default parameters for the decomposition of sensor data: spherical harmonics of order 8 and 3 for the inner and outer component, respectively. The segment length was chosen to be 10 ​s without overlap. In case of Elekta Neuromag, we used the MaxFilter software with the same settings. In accordance with Medvedovsky et al. (2009), the correlation limit (*CL)* was varied between 0.95–0.60.

### 2.4 Evaluation of the results

We evaluated the results both at the sensor and source level in the frequency domain. The power spectrum was calculated in Brainstorm with 4 ​s Hamming windows and 50% overlap using Welch’s method (Welch, 1967).

#### 2.4.1 Sensor level comparison

To determine the effectiveness of the four different cleaning algorithms we calculated the root mean squared error (RMSE) of the logarithmic power between the reference recording and the cleaned version of the DSMW recording over the various channels. This measures how far the resulting cleaned sensor-level data on average deviates from the ground truth (under a quadratic loss function). We compute the RMSE separately for the average power in three different frequency ranges: the dipole frequency (11.5–12.5 ​Hz), the DBS frequency (peak ​± ​1Hz; peak CTF: 130.5 ​Hz, peak Elekta Neuromag: 127.75 ​Hz), and in the low-frequency movement-related range (1–15 ​Hz, excluding 11.5–12.5 ​Hz). Formally:

$$RMSE\;\left(band\right)=\sqrt{\frac{1}{N}\sum_{i=1}^{N}{\left(\frac{\pi_{i}^{DSMW_{cleaned}}\left(band\right)-\pi_{i}^{ref}\left(band\right)}{\pi_{i}^{ref}\left(band\right)}\right)}^{2}}$$

Where N denotes the number of artefact-free channels (273 in CTF, 293 in Elekta Neuromag recordings), π(band) denotes the averaged log power in the respective frequency band∈{[1–11.5 12.5–15], [11.5–12.5], [129.5–131.5] for CTF and [126.75–128.75] for Elekta Neuromag} and the superscripts ref and DSMW_cleaned_ denote the reference condition and the cleaned DSMW condition, respectively.

#### 2.4.2 Source level comparison

A forward model for the phantom was created using available functions in Brainstorm. The source space was defined with a 5 ​mm spaced grid inside the phantom model leading to 10,039 voxels for the CTF phantom and 16,430 for the Elekta Neuromag phantom. The lead field for CTF was calculated using the overlapping spheres method (Huang et al., 1999) in Brainstorm. A forward model for magnetic dipoles was not available in Brainstorm. Therefore, for Elekta Neuromag recordings, we used a single-shell realistic head model (Nolte, 2003) in Fieldtrip. We used Linearly Constrained Minimum Variance (LCMV) beamforming with default settings in Brainstorm to obtain source time series (Van Veen et al., 1997). The data covariance matrix was obtained from the sensor data of each recording as well as the cleaned data separately and regularized by replacing its smaller eigenvalues with the median value through available Brainstorm functions. We used the empty room recording to calculate the noise covariance matrix.

We evaluated the active sources of the reference recording and compared them to those of DSMW and cleaned recordings at 12 ​Hz. Active sources for each dataset were determined using a threshold value based on bootstrapping (Efron, 1979). Bootstrapping was applied on the windowed power estimates used previously to calculate Welch’s power spectrum. We averaged randomly chosen windows (104 in CTF, 54 in Elekta Neuromag recordings) with replacement for each source, excluding the sources which were active in the reference recording. This process was repeated 1000 times for each dataset. The 95th percentile of power values across all source locations and all iterations was defined as the significance threshold. To determine the spatial overlap between the reference recording and the (cleaned) DSMW version we used the D measure (Mesmoudi et al., 2013):

$$D\left(Reference,\;DSMW\right)=\frac{Reference\cap DSMW}{Reference\cup DSMW}\;.$$

The overlap is calculated across all sources. If there is a perfect spatial overlap between the Reference and DSMW, D will be 1.

#### 2.4.3 Optimal parameter choice for the different artefact rejection algorithms

Each artefact rejection algorithm has one parameter that governs the strength of cleaning. At the sensor level, we selected the optimal parameter based on the lowest average RMSE across dipole, movement, and DBS frequencies. In case of source level, starting from the respective initial values reported above, we increased each parameter as long as there was still an improvement larger than 0.01 in D.

## 3 Results

### 3.1 Sensor level

**Fig. 1:**
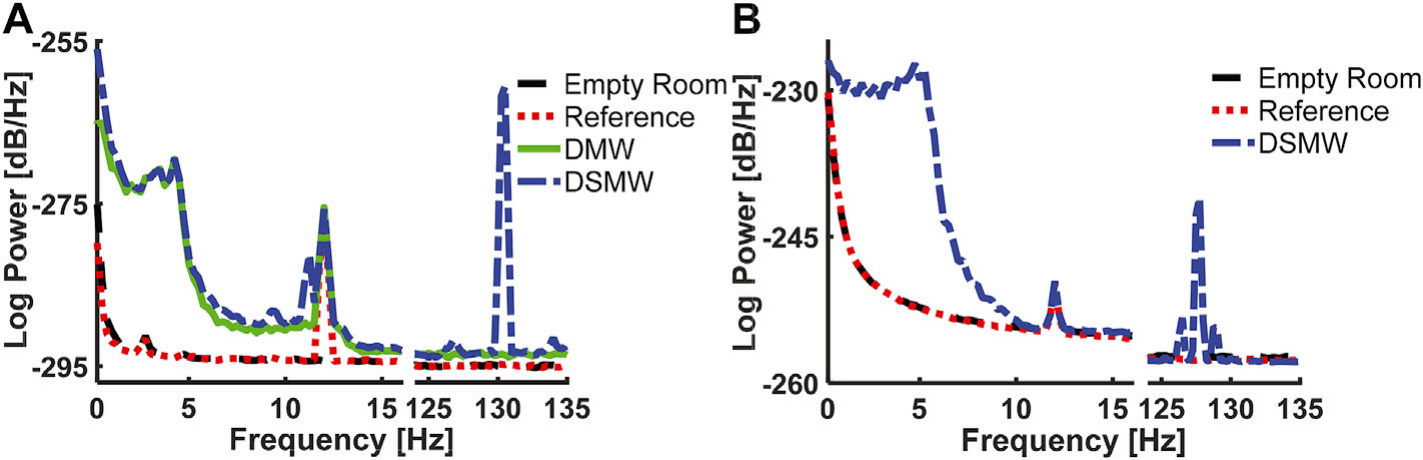
Power spectra of the phantom measurements. A: CTF recordings; B: Elekta Neuromag recordings. The power spectra of each measurement are averaged across channels and reported for the range of [0 ​Hz,15 ​Hz] in the left panel and for [125Hz, 135 ​Hz] in the right panel. Movement and wire add wide-band noise below 15 ​Hz and 10 ​Hz in CTF and Elekta Neuromag recordings, respectively. DBS stimulation introduces an artefact around the stimulation frequency of 130 ​Hz. In an ideal scenario, a cleaning algorithm would remove both types of artefact while preserving the dipole activity at 12 ​Hz, i.e. correspond to the reference recording.

We first examined the spectral features of the recordings (see Fig. 1). We separated the spectrum into two parts. In the left panel, we consider the 0–15 ​Hz range because a lot of physiologically-relevant brain activity is found in this low-frequency range that is most affected by movement and wire artefacts. The right panel focuses on the range of 125–135 ​Hz. That range should exhibit a clear DBS artefact while potentially also containing physiologically relevant signals (Florin and Baillet, 2015; Ross et al., 2020).

**Fig. 2:**
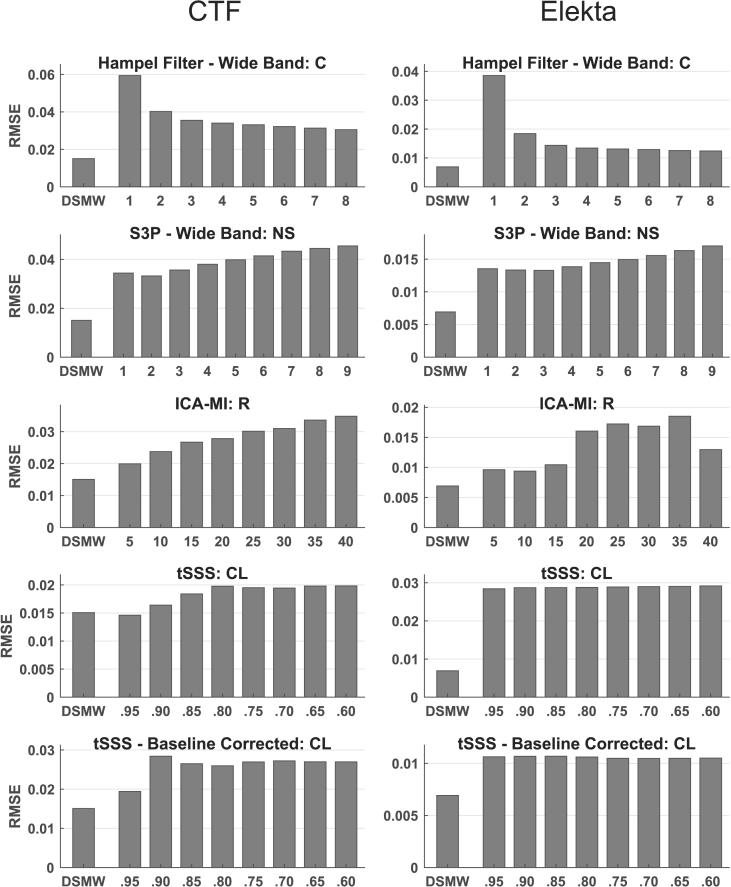
RMSE for the dipole frequency (11.5–12.5 ​Hz) relative to reference recording for the different cleaning algorithms. Left panel: CTF recordings; right panel: Elekta Neuromag recordings. The first bar represents the RMSE of the uncleaned DSMW recording compared to the reference recording. The other bar groups show the RMSE of the respective artefact cleaning technique with different parameterizations. None of the methods improved the results at dipole frequency (R: Rejection rate, NS: Noise subspace, C: Constant, CL: Correlation limit; WB: wide band; BL: baseline corrected).

**Fig. 3:**
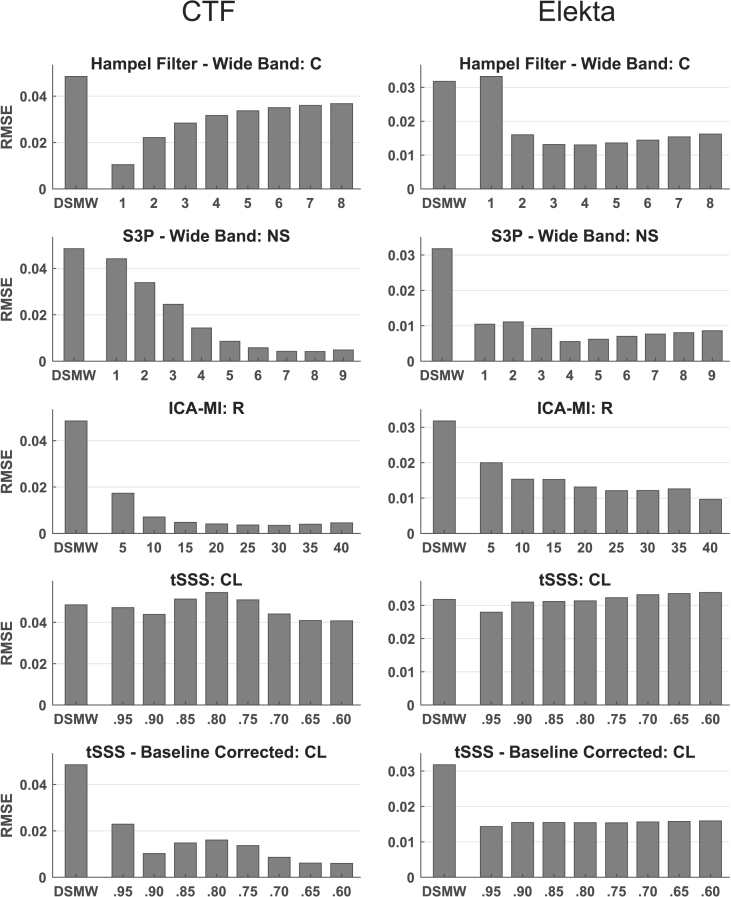
RMSE at movement frequency (1–15 ​Hz, excluding 11.5–12.5Hz) relative to reference recording for the different cleaning algorithms. Left panel: CTF recordings; right panel: Elekta Neuromag recordings. ICA-MI performed the best in CTF recordings while the lowest RMSE in Elekta Neuromag recordings was achieved with WB S3P (R: Rejection rate, NS: Noise subspace, C: Constant, CL: Correlation limit; WB: wide band; BL: baseline corrected).

**Fig. 4:**
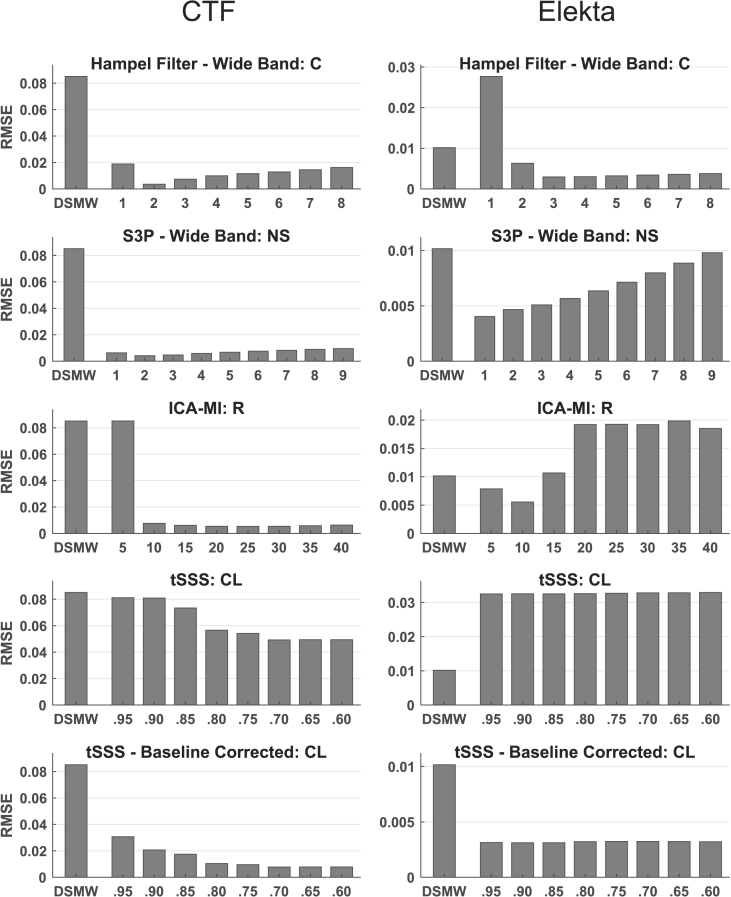
RMSE for DBS frequency relative to reference recording for the different cleaning algorithms. Left panel: CTF recordings with frequency range 129.5–131.5 ​Hz; right panel: Elekta Neuromag recordings with frequency range 126.75–128.75 ​Hz. ICA-MI and the Hampel filter performed well in both MEG systems (R: Rejection rate, NS: Noise subspace, C: Constant, CL: Correlation limit; WB: wide band; BL: baseline corrected).

Fig. 2, Fig. 3, Fig. 4 show the root mean squared error (RMSE) between the reference recording and the cleaned versions of the DSMW after the artefact-rejection methods have been applied for dipole (11.5–12.5 ​Hz), movement (1–15 ​Hz, excluding 11.5–12.5 ​Hz), and DBS frequencies (129.5–131.5 ​Hz for CTF, 126.75–128.75 ​Hz for Elekta Neuromag), respectively. In each figure, the left panel reports the results for the CTF data and the right panel the results for the Elekta Neuromag recordings. Each subgraph groups the different parameterizations of the respective artefact-rejection methods.

**Fig. 5:**
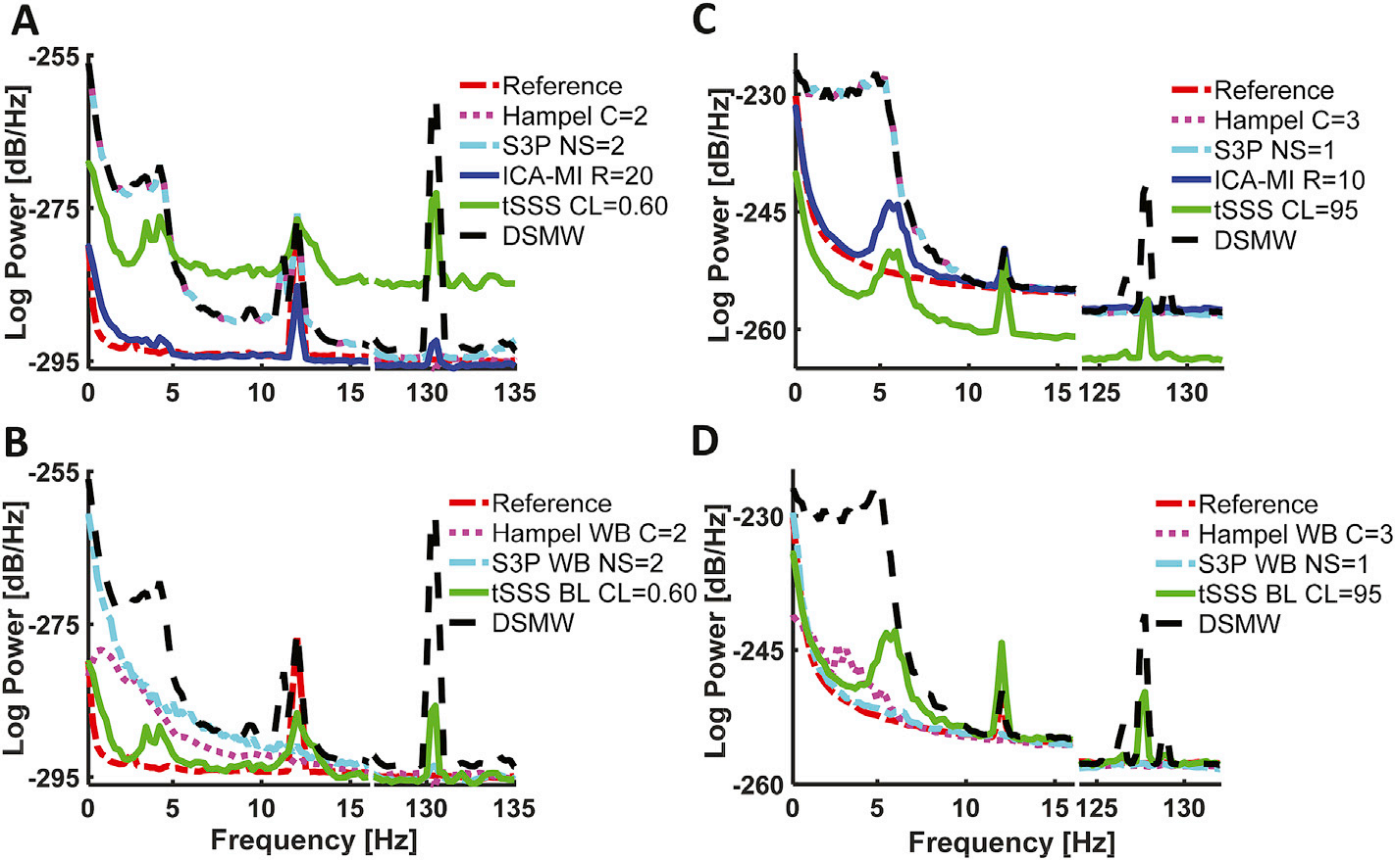
Power spectra for the different artefact rejection methods under optimal parameter settings. A, B: CTF recordings; C, D: Elekta Neuromag recordings. The power spectra of each measurement after artefact removal were averaged across channels and are reported for the range of [0 ​Hz, 15 ​Hz] and [125Hz, 135 ​Hz]. Power spectra of the cleaning algorithms using their originally recommended setting are provided in the top panel. Note the shift in baseline level for tSSS (upward in CTF, downward in Elekta Neuromag recordings, respectively). Power spectra of the cleaning algorithms after changing them from the originally recommended usage are provided in the bottom panel. For tSSS the removal of movement-related artefacts improves, the Hampel filter and S3P now remove parts of the movement-related artefact, but also the dipole activity.

Fig. 5 illustrates the effect in the frequency domain for each method with its best parameter setting.

#### 3.1.1 CTF results

The empty room recording (black dashed line) captures the overall noise level of the system (see Fig. 1A). The ‘Reference’ condition (red dotted line), which simply is an empty room recording with the dipole at 12 ​Hz turned on, showed very similar activity, but with the expected clear peak at 12 ​Hz. This reference recording is taken as the ground truth for all further comparisons. The DMW recording (green line) adds phantom movement to this as well as an attached wire. This introduced a clear increase in power below 15 ​Hz compared to the reference recording, with the strongest increase below 5 ​Hz. Finally, the figure depicts the DSMW recording with all noise sources (DBS stimulator, movement, and wires) present simultaneously in addition to the dipole being activated. As expected, the DSMW power spectrum (blue dashed line) combines the individual effects: wide-band noise in particular below 5 ​Hz and a peak at 12 ​Hz. In addition to the DBS frequency, the DSMW recording showed peaks at aliased frequencies (Supplementary Fig. 2).

There are three main takeaways from the RMSE results. First, focusing only on the dipole frequency in Fig. 2 (left panel), it becomes apparent that none of the methods achieved a better RMSE than the DSMW, i.e. the DBS artefact rejection methods were unable to improve the signal-to-noise ratio in this frequency range compared to the untreated data. Second, when comparing Fig. 2 with Fig. 3, Fig. 4 there is a trade-off for all methods between preserving the dipole activity and removing most of both the DBS artefact at 130 ​Hz and the movement-related artefact. Removing more of the artefacts typically also involves removing some signal. Third, the performance of cleaning algorithms was relatively insensitive to the respective parameter settings. Generally, once a certain level of artefact rejection was reached, stronger settings yielded no further improvement.

In addition to only applying S3P and a Hampel filter to the DBS artefact, we also applied S3P and Hampel filter to the whole spectrum. The rationale is that an application only to the DBS frequency would not affect the movement-related artefact at all and we wanted to know whether the two techniques could also be used to alleviate this artefact. Fig. 2, Fig. 3 illustrate the wide-band (WB) versions of Hampel filter and S3P. As shown in Fig. 3 both filters are indeed able to partially remove the movement-related artefact. But this comes at the cost of also removing most of the actual dipole activity (see Fig. 2). Fig. 4 shows that S3P performs best across methods in removing the artefact at the DBS frequency.

In terms of overall performance, ICA-MI with a 20% rejection rate had the lowest average RMSE across all three frequency bands (movement, dipole, and DBS). When removing the DBS artefact, its performance was almost on par with the one of S3P, while at the same time removing the movement-related artefact quite well and simultaneously preserving the dipole activity. The low RMSE of ICA-MI is also reflected in the power spectrum (see Fig. 5A). In contrast, tSSS did not provide a satisfactory removal of the DBS and movement artefact. It only performed well in preserving the dipole activity. The reason for this is a pronounced upward shift of baseline power levels. This can be clearly seen from the power spectra of the data after artefact removal (Fig. 5A). In order to also provide results that correct for this baseline shift, we first calculated for each channel the difference in average logarithmic power between the cleaned and reference recording from 15 to 125 ​Hz (excluding the line noise at 50 and 100 ​Hz). This value was then subtracted from the cleaned data on a channel by channel basis. After this correction, the results of tSSS are more comparable to the other methods (see Fig. 5B). The RMSE improved for the movement-related artefact removal and the DBS artefact removal, while at the same time increasing for the dipole activity range.

#### 3.1.2 Elekta Neuromag results

The phantom recording with the Elekta Neuromag system exhibited properties similar to the CTF system, i.e. movement added low-frequency components to the reference recording (see Fig. 1B) while the 12 ​Hz dipole activity was preserved. Interestingly, no aliased frequencies were found for the Elekta Neuromag recording (see Supplementary Fig. 3).

Similar to the CTF cleaning results, none of the methods achieved a better RMSE than the DSMW at the dipole frequency (see Fig. 2 right panel). DBS noise suppression for the Elekta Neuromag recordings at DBS frequency was not as high as for the CTF recordings (see Fig. 4). This difference is due to the fact that for the Elekta Neuromag system around 50% of the channels were affected by the stimulation artefact, whereas ~95% of channels showed a clear peak at the DBS frequency in CTF recordings (see Supplementary Fig. 4). Overall, ICA-MI (R ​= ​10) performed better than the other methods in suppressing movement and DBS artefacts while preserving the dipole. This is also seen in the frequency spectrum (see Fig. 5C). The performance of the baseline-corrected version of tSSS was comparable to ICA-MI (see Fig. 5D). As expected, when applied to the whole spectrum the Hampel filter and S3P suppressed the dipole activity along with DBS and movement artefacts. Contrary to the CTF results, tSSS shifted the average power spectrum downward. However, this downward shift is not evident in every individual channel (see Supplementary Fig. 5). tSSS introduces high between-channel variability: Some of the channels display an extensive power drop, while power remained the same in the other channels. Although dipole power was increased after cleaning with tSSS, the DBS artefact was still clearly visible in the power spectrum.

#### 3.1.3 Robustness of methods to parametrization

In case of real data recordings, the ground truth is unknown. Therefore, wrong parametrization may lead to either insufficient noise suppression or removal of intrinsic brain activity. For this reason, robustness of a noise suppression method is essential. We evaluated the robustness of the methods to parametrization based on RMSE variation in the applied parameter range (see Table 1). For CTF recordings, ICA-MI and S3P showed similarly low variation and therefore robustness to suboptimal parameter choices, followed by the Hampel filter. tSSS displayed the largest performance variation within the tested parameter range. For Elekta Neuromag recordings, however, tSSS and the Hampel filter showed the lowest variation followed by S3P and ICA-MI.

**Table 1:** RMSE variation over the parameter space of the cleaning methods. Note that the RMSE is for the DBS frequency only in case of the Hampel filter and S3P. For ICA-MI and tSSS it is calculated for the movement, dipole, and DBS frequency ranges.

Cleaning Method	CTF Recordings	Elekta Neuromag Recordings
Hampel filter (C ​= ​2–8)	0.0108 ​± ​0.0043	0.00375 ​± ​0.00116
S3P (NS ​= ​1–9)	0.00689 ​± ​0.00183	0.00662 ​± ​0.00197
ICA-MI (R ​= ​10–40)	0.0134 ​± ​0.0011	0.0145 ​± ​0.0026
tSSS (CL ​= ​0.95–0.60)	0.0423 ​± ​0.0049	0.0311 ​± ​0.0008

### 3.2 Source level

To evaluate the effectiveness of the artefact rejection methods in the source space, we examined spatial activation patterns. To this end, we obtained active sources by thresholding source maps at 12 ​Hz with critical values at the 95th percentile of the bootstrapped source maps (see methods).

#### 3.2.1 CTF results

**Fig. 6:**
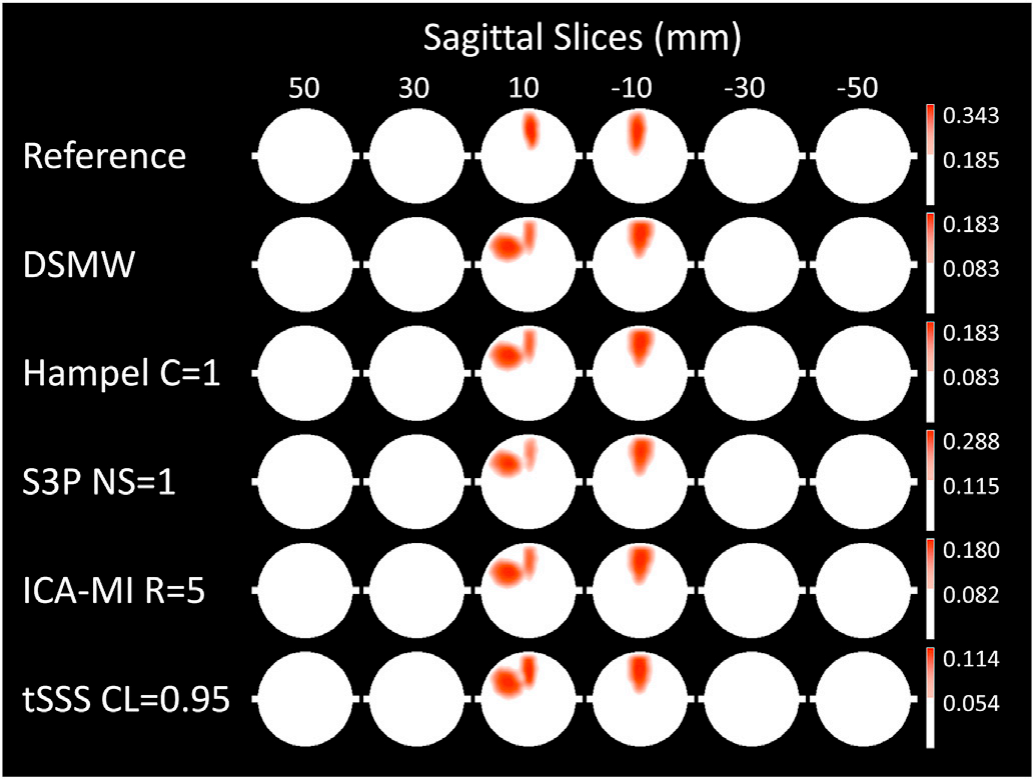
Significantly active sources at the frequency of the dipole (12 ​Hz), CTF recordings. Sagittal slices covering more than 70% of total phantom volume are used to visualize source activity. Slices outside the covered volume do not reveal any activity. The first row with the reference recording displays the activation one would expect after successful artefact removal. Activation of DBS introduces spatial leakage at the frequency of interest. None of the methods completely removed additional activation while preserving the dipole activation. The activation pattern closest to the reference condition was achieved by tSSS. The colorbar indicates the power values, thresholded at the 95% percentile.

**Fig. 7:**
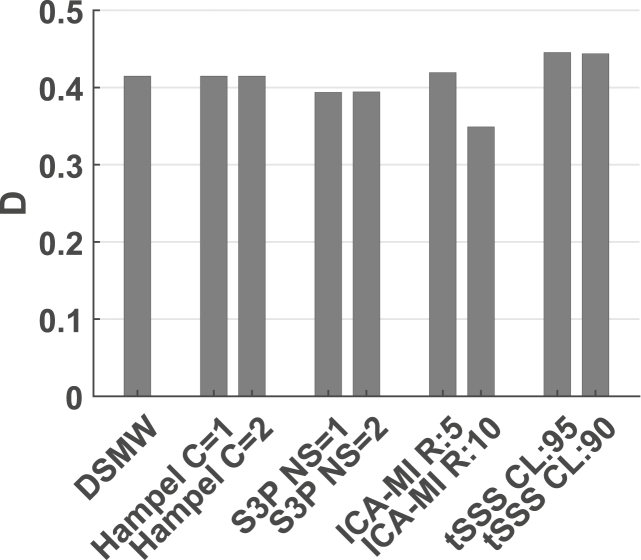
D for the different artefact rejection methods, CTF recordings. Correspondence between active and non-active sources of the reference recording and the cleaned recording conditions. A higher D denotes a better overlap. Note that only tSSS improved the correspondence to the reference recording.

Significant sources are depicted in Fig. 6. For the reference recording the active dipole is located in central frontal regions across the heights from 10 to −10 mm. This activation pattern is subsequently taken as the baseline to evaluate the performance of the cleaning algorithms in the source space. Compared to the activations in the reference recording, the DSMW condition presents additional active regions. None of the artefact-rejection methods was completely successful in removing these spurious activations completely without also removing parts of the dipole activity. Based on D, tSSS (CL ​= ​0.95) cleaned and source-reconstructed data revealed the highest overlap with the reference recording (Fig. 7). This was followed by ICA-MI (R ​= ​5%), Hampel (C ​= ​1), and S3P (NS ​= ​1). Note that only tSSS achieved a better correspondence with the reference recording than the uncleaned DSMW data. Although all methods preserved the dipole’s original location, some reduced its spatial extent and none of the methods removed the artefact-related additional spurious sources (see Fig. 6).

**Fig. 8:**
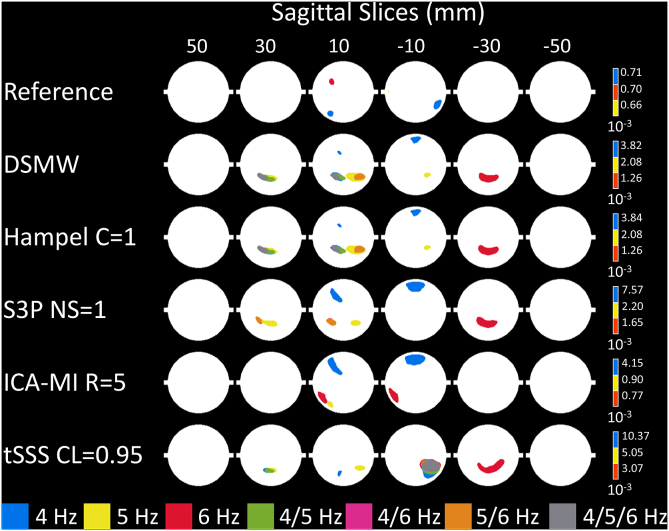
Significantly active sources related to movement of the wire at 4, 5, 6 ​Hz, CTF recordings. Sagittal slices covering more than 70% of total phantom volume are used to visualize source activity. The color bar represents highest power values at the relevant frequencies. None of the methods managed to remove movement-related activations completely.

In addition, we evaluated the activity at the frequencies mostly affected (4, 5, 6 ​Hz) by movement and wire in a similar fashion. Significantly active sources color-coded according to the frequency are illustrated in Fig. 8. In case a source was active at 2 or all 3 frequencies we color-coded these frequencies with a different color (see figure legend). As we chose the 95th percentile as the threshold, there are still a few active regions in the reference recording. However, these are most likely due to false positives. When moving to the DSMW case, the overall power level is increased by a factor of ~5 across these 3 frequencies. The most prominent increases are in the posterior regions where the wire was taped to the phantom. None of the cleaning algorithms was able to bring the power level to the one of the reference recording, and therefore, active sources are found throughout the source space.

#### 3.2.2 Elekta Neuromag results

**Fig. 9:**
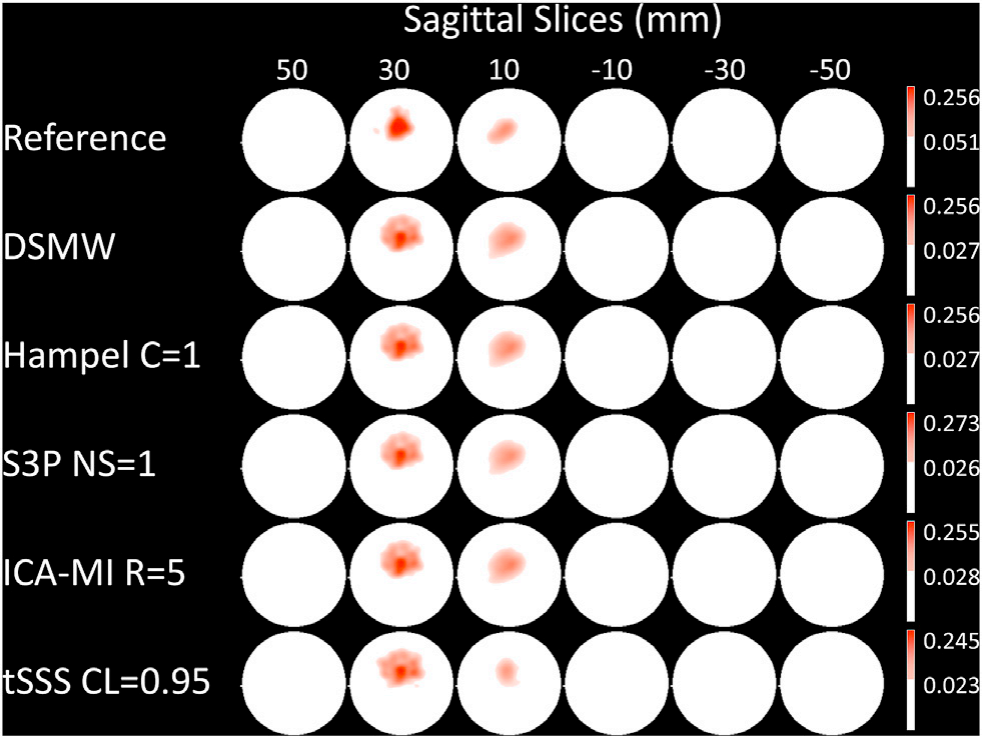
Significantly active sources at the dipole frequency (12 ​Hz), Elekta Neuromag recordings. Sagittal slices covering more than 70% of total phantom volume are used to visualize source activity. Slices outside the covered volume do not reveal any activity. The first row with the reference recording displays the activation one would expect after successful artefact removal. Activation of DBS introduces spreading of the activity at 12 ​Hz. None of the methods managed to restore reference activity. The colorbar indicates the power values, thresholded at the 95% percentile.

**Fig. 10:**
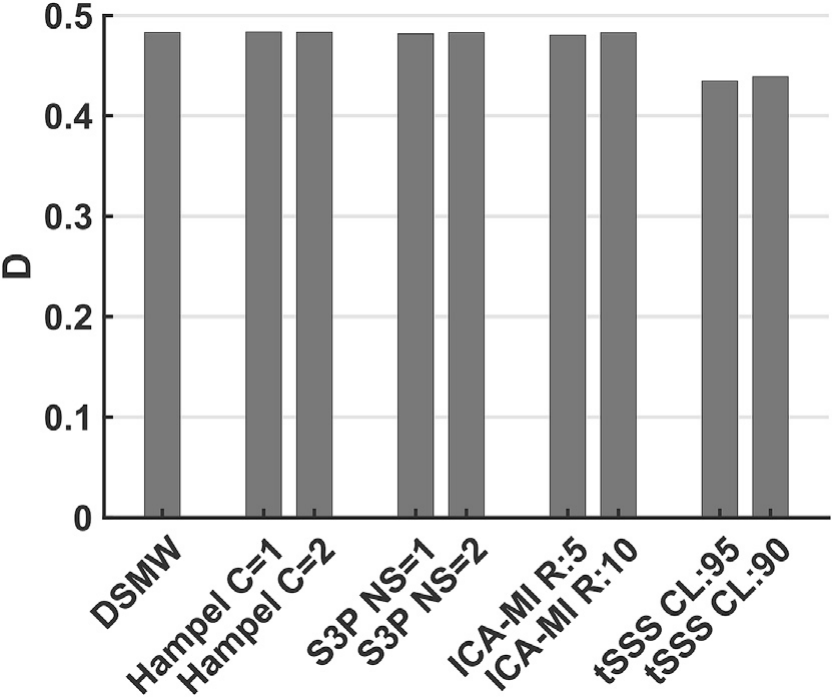
D for the different artefact rejection methods, Elekta Neuromag recordings. Correspondence between active and non-active sources of the reference recording and the cleaned recording conditions. A higher D denotes a better overlap. None of the methods managed to increase the D.

Compared to the CTF recordings, the source activity at 12 ​Hz was more spread out for the DSMW obtained with the Elekta Neuromag recording (see Fig. 9). Only tSSS was able to reduce this source spread to a level similar to the reference recording. However, D indicates that the correspondence with the reference recording was actually lower (Fig. 10). Therefore, none of the cleaning algorithms increased the amount of overlap between the reference recording and DSMW.

**Fig. 11:**
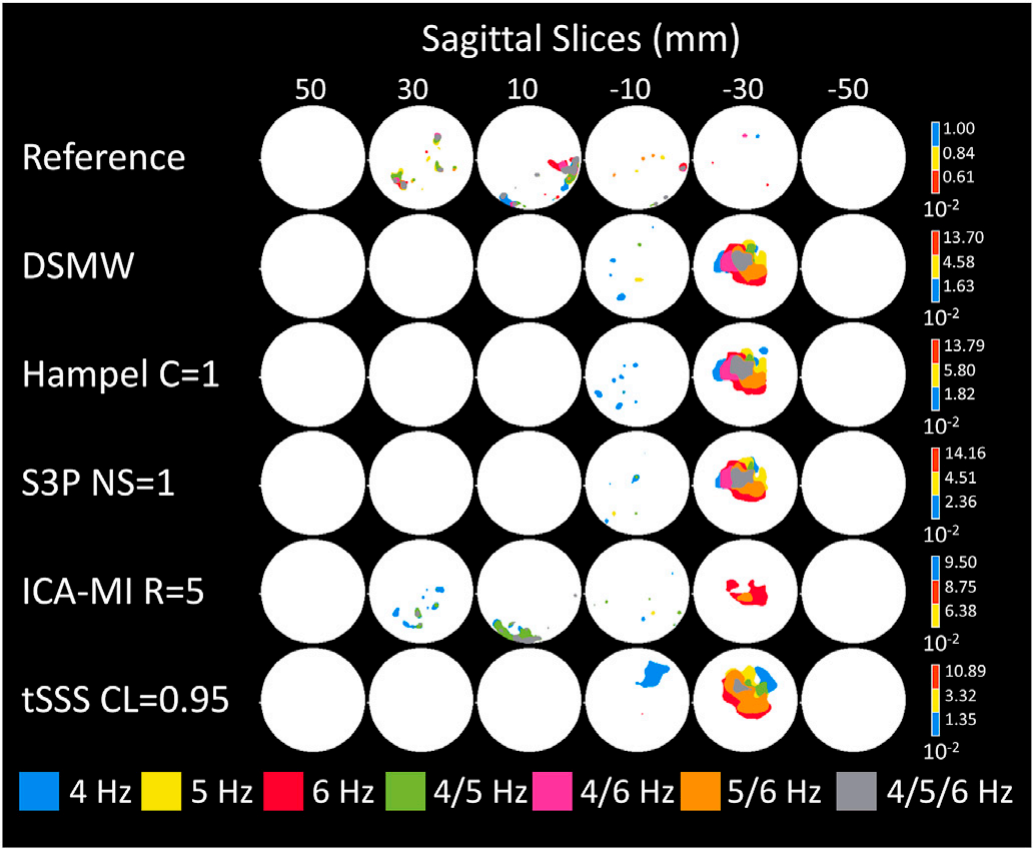
Significantly active sources related to movement of the wire at 4, 5, 6 ​Hz, Elekta Neuromag recordings. Sagittal slices covering more than 70% of total phantom volume are used to visualize source activity. The color bar represents highest power values at the relevant frequencies. None of the methods managed to remove movement-related activations completely.

Similar to CTF recordings, movement induced false activations (see Fig. 11). None of the methods suppressed this activation completely. Nevertheless, compared to other methods, ICA-MI reduced the movement-related activations at the source level.

## 4 Discussion

We performed a MEG phantom study under realistic DBS stimulation conditions to identify artefacts introduced by DBS and to evaluate the performance of different DBS artefact rejection methods previously employed in the literature. At the sensor level, besides the expected DBS-related artefact at 130 ​Hz, movement caused low-frequency artefacts due to the extension wires. When moving to the source level, DBS stimulation introduced false activations at the dipole frequency and lower frequencies due to movement. Considering the different cleaning algorithms ICA-MI outperformed the other methods at suppressing DBS and movement-related artefacts at the sensor level while preserving the “true” dipole activity. Conversely, tSSS revealed a better spatial source map with more resemblance to that of the reference recording.

Although ICA-MI performed well at the sensor level, it did not yield the best results at the source level, but tSSS. This contradiction could be stemming from a disproportional signal rejection with ICA-MI from sensor level data, i.e. in the case of source level data 5% of the data were removed. Alternatively, the reference signal containing residual information about the dipole activity could be causing an undesirable rejection of informative components. On the other hand, the poor performance at the sensor level of tSSS is due to its baseline shift (upwards in CTF, downwards in Elekta Neuromag). Therefore, we also evaluated a baseline-corrected version for tSSS. This adjustment decreased overall RMSE in both CTF and Elekta Neuromag recordings, bringing its performance to a level similar to ICA-MI.

S3P is designed for applications to a pre-specified frequency range and the Hampel filter for removing narrow frequency peaks. Therefore, applying both filters to only the DBS frequency peak would be the natural choice. However, if they are applied to only the DBS frequency range, they are unable to remove any of the movement-related artefact outside of this range. On the other hand, when applying them to the wide-band signal, they remove the movement-related artefact to a certain extent while at the same time removing the dipole activity. In our phantom recording, the dipole activity resembles a sharp frequency peak, whereas intrinsic brain activity usually produces a smoother peak. Therefore, the actual dipole peak was also classified as an outlier by the Hampel filter and removed. But even if one considers this not to be a realistic scenario, the Hampel filter was also not able to remove the movement-related artefact in a satisfying way. The reason is that the movement-related power increase is a smooth increase in power and no outlier is detected by the Hampel filter. In case of S3P, it is explicitly indicated by the authors that the segments with high SNR should not be processed by the method. This approach was impractical in case of our phantom recording because the DBS artefact - in particular in the presence of movement - contaminates most of the frequency spectrum. This issue would, however, also occur in most real recordings, because the DBS artefact and in particular the movement-related artefacts, which mimicked human pulsation, are in the frequency range of interest of most studies.

### 4.1 Choice of parameters

In the following we will provide recommendations on how to choose the optimal parameter range for each cleaning algorithm, because in real data the ground truth is usually unknown. In case of the Hampel filter, it is advisable to determine the optimal value visually from the spectrum of the cleaned DBS signal. The most conservative cleaning with C ​= ​1 leads to an overcompensation of the DBS artefact and is therefore not recommended. Values larger than 1 lead to good cleaning with little variation in case of misestimating in the range from 2 to 8. S3P is quite robust to changes in NS, i.e. there is only little risk from overestimating the noise subspace in a sensible range. However, aiming to remove as little signal as possible from the data suggests to use low NS. If one only applies S3P to the DBS frequency itself, one can confirm its performance also visually based on the cleaned spectrum. In case of ICA-MI, there is a trade of between the amount of noise suppression (movement or DBS) and brain activity preservation. The best removal of the DBS artefact was achieved with a rejection rate of 10–15%, while for the movement related artefact even more components needed to be removed (30–40%). Of note here is that removing more components resulted only in minor cleaning improvement of the movement related noise, while the RMSE increased substantially at the dipole frequency. As deviations from the optimal rejection rate do not cause quantitatively big performance losses, a low as possible rejection rate should be used and confirmed by the cleaning of the DBS-artefact from the spectrum. In the case of tSSS, the dipole activation is not affected greatly by the decrease in correlation limit. We therefore recommend to aim for a good removal of the DBS related artefact, which is straightforward to verify, because the movement related artefacts were then suppressed as well. In the case of the phantom measurements, an optimal DBS removal was achieved with a correlation limit of 0.95 for the Elekta Neuromag recording and 0.7 for the CTF recording.

### 4.2 Comparison to previous literature

A previous study with the same phantom investigated the coherence between MEG and a reference channel in the presence of DBS artefacts (Oswal et al., 2016). In that study, a copy of the sinusoidal signal driving the dipolar source was recorded with an external amplifier system. Dynamic imaging of coherent sources (DICS; Gross et al., 2001) was used to find the location of peak coherence between the recorded copy of the dipole activity and MEG source level data. Later LCMV (Van Veen et al., 1997) beamforming was used to extract time series of the peak coherence location for a coherence analysis. The DBS ON and DBS OFF conditions showed comparable coherence results and it was concluded that beamforming is effective in suppressing DBS-related artefacts at the source level. However, the analysis was limited to coherence. Although the effect of the DBS artefact on peak coherence location is seemingly negligible, this result is not transferable to other measures such as power. The present study demonstrates that DBS-related artefacts induce false activations at the source level despite the utilization of LCMV beamforming. This finding suggests that beamforming alone is insufficient to suppress DBS artefacts and an artefact rejection method is required prior to analysis.

Litvak et al. (2010) and Oswal et al. (2016) previously reported strong artefacts caused by ferromagnetic extension wires used in externalized patients in which the electrodes have been implanted, but the generator is not yet implanted. They suggested that the artefacts stem from percutaneous ferromagnetic wires which are replaced with non-ferromagnetic wires once the DBS stimulator is implanted. In the present study we used extension wires that are used for implantation and should be non-ferromagnetic, because those are implanted in patients with the complete DBS system. Nevertheless, in both DMW and DSMW recordings the movement in combination with the wires had a clear effect on low frequencies. Therefore, artefacts arising solely from the moving wires can contaminate MEG measurements even if DBS is turned off.

To our knowledge, this paper presents the first systematic comparison between available DBS artefact rejection methods for MEG. Previously, Lio et al. (2018) conducted a systematic literature review of EEG studies using various DBS artefact rejection methods. Discussed methods included analog and digital filters as well as more advanced methods operating in the frequency domain. Based on simulations where they analyzed aliased frequencies the authors concluded that no single approach could effectively remove DBS artefacts. However, a combination of filters was suggested as an effective alternative solution. Still, the problematic issue of movement-related artefacts could not be addressed, because with EEG a phantom study is hardly possible.

More recently, Boring et al. (2019) employed machine learning techniques to quantitatively compare the amount of recovered information from the recordings with DBS ON and DBS OFF in a visual search task. Signal-space projection (SSP; Tesche et al., 1995; Uusitalo and Ilmoniemi, 1997), 1–50 ​Hz band-pass filter, tSSS, and principal component analysis (PCA) were applied in consecutive order. After each step, multivariate pattern analysis was used on the preprocessed data to predict the category of the stimuli. DBS ON and DBS OFF conditions showed comparable results in categorizing the stimuli and each pre-processing step increased the classification accuracy. Based on this result the authors concluded that tSSS sufficiently removed the DBS artefact. It should be noted, however, that the main outcome measure of this study was the classification accuracy in the visual search task. As classification success only requires recovering some form of predictable pattern underlying the binary outcome, it is not clear how well the DBS-related artefacts were actually removed and how this finding generalizes to other analysis techniques and objects of interest.

In addition to the methods evaluated in the present paper, several other approaches for DBS artefact rejection were introduced over the last years. Santillán-Guzmán et al. (2013) proposed a time-frequency domain filter to suppress DBS artefacts at the frequency of stimulation and its harmonics. The filter is based on estimating the phase, frequency, and amplitude of the artefact. The estimated artefact is later subtracted from the recordings in the time domain. Similarly, Sun et al. (2014) suggested applying matched filters at the DBS frequency and its aliased frequencies. More recently, another temporal domain-based approach revealed that subtraction of DBS signal templates from the recordings produces satisfying results (Sun and Hinrichs, 2016). Although these methods are proven to be effective in suppressing the artefacts arising from the stimulation signal, artefacts at lower frequencies due to wires and movement are not addressed. For that reason, these methods were not tested in the present study. Moreover, we only evaluated methods which suppress artefacts at the sensor level. The rationale for this choice was to give the researcher flexibility in choosing between various analysis methods by obtaining artefact-free sensor level data. Accordingly, we also did not evaluate null-beamformer (Mohseni et al., 2010), which successfully suppresses DBS artefacts around the burr holes by placing a null at its location during source reconstruction.

More recently, a new algorithm exploiting sensitivity differences between planar gradiometers and magnetometers was proposed by Samuelsson et al. (2019). Although potentially useful, we could not include this method in our study because the algorithm is system-dependent and currently can only be applied to recordings conducted with Elekta Neuromag. The effectiveness of this method should be evaluated in future studies.

### 4.3 Methodological considerations and limitations

In the present study, RMSE is used to evaluate the success of the cleaning algorithms. We applied 3rd order gradient noise compensation for the CTF recordings and SSPs for the Elekta Neuromag recordings to suppress environmental noise. tSSS is known to potentially reduce environmental noise (Airaksinen et al., 2011; Gonzalez-Moreno et al., 2014). If this caused the noise level to decrease below the one of the empty room recording, the resulting RMSE could be misleading for evaluating the performance of artefact removal. We did not find this to be the case for CTF recordings, as seen in Fig. 5. However, for the Elekta Neuromag recording the environmental noise was on average further reduced across channels. This was associated with great variability in baseline level at the single channel level (see Supplementary Fig. 5). Therefore, it is not possible to ascertain a uniform noise reduction across channels, resulting in incomparable power across sensors.

We generated movement-related artefacts by placing a pulsating mechanism under the phantom. The movement mechanism had only one degree of freedom, i.e., the phantom movement was limited to the vertical axis. In patient recordings, although arterial pulsations have similar movement patterns to the phantom movements in the present study, head movements are likely to create more complex artefacts. The success of the discussed methods in cleaning movement artefacts depends heavily on the type and complexity of the movement. Therefore, our results may not generalize to every type of movement artefact in patient recordings. Moreover, the phantom did not contain a DBS generator, which could generate further artefacts in patient recordings. The extent of artefacts introduced by the DBS generator highly depends on the location of the implanted generator. In general, because the generator is below the head (chest or abdomen), spatial artefact-removal techniques should be able to clean those artefacts. For a more realistic setting, future phantom investigations should include a generator placed at a similar distance as in patients. Still, our results indicate that despite the presence of a relatively simple movement, the current algorithms were not able to remove the arising artefact.

In the present study, we used a single dipole oscillating at 12 ​Hz to mimic brain activity. In patient recordings, multiple sources contribute to the recorded signal at various frequencies. In such a scenario, the efficacy of the methods discussed here could differ. For example, due to there being a narrow peak in the current setup, the Hampel filter removes the dipole activity if applied to the whole spectrum. In patient recordings, source activity from multiple sources could spread to adjacent frequencies and would prevent the occurrence of narrow peaks at lower frequencies. Consequently, the dipole activity would potentially not be detected by the Hampel identifier and therefore not be removed. Furthermore, ICA and tSSS could actually benefit from being able to rely on multiple brain signals to distinguish between noise and brain data. Future studies should consider multiple dipole activations to investigate this effect.

As stated above, in patient recordings the reference signal in ICA-MI is measured using surface electrodes placed on the implanted stimulator. In the present study, however, the reference signal was recorded from inside the phantom, which could have led to more signal information being time-locked to the movement. In patient recordings the surface electrodes might not pick up head movements. Therefore, the mutual information between the reference signal and the MEG channels could be more accurate in the present study than in patient recordings.

Although band-pass filtering was not considered in this study, it is often a standard preprocessing step in many MEG studies. S3P and the Hampel filter should not be affected by filtering because they operate in the frequency domain. tSSS and ICA-MI are time domain operations and for tSSS the guideline is to apply it before filtering. For ICA-MI, applying a low-pass filter first led to a suboptimal identification of the movement artefact, because the movement artefact was coupled to the DBS artefact and low-pass filtering removed this information. Therefore, ICA-MI and tSSS should be applied before filtering the data.

Lastly, we would like to point out that tSSS relies heavily on the sensor position and their orientation. Due to different manufacturing processes, the information about the sensor location for Elekta Neuromag systems is more precise than for CTF systems. Still, tSSS did not yield better cleaning results for the Elekta Neuromag recordings. While the baseline shift on average does seem smaller for the Elekta Neuromag system, the variance in the baseline shift between channels is larger. As a result, the RMSE was similar for the Elekta Neuromag and CTF system.

In conclusion, DBS artefacts contaminate the power spectrum at multiple frequencies and introduce spatial leakage at dipole frequency in source space. LCMV beamforming was not sufficient to suppress false activations introduced by DBS and none of the methods managed to remove the artefacts perfectly. ICA-MI and tSSS showed good results at the sensor level while tSSS performed better at the source level. Therefore, at the moment these are the methods of choice when combining MEG recordings with DBS. Future studies should invest into finding even better cleaning algorithms.

## CRediT authorship contribution statement

**Ahmet Levent Kandemir:** Data curation, Formal analysis, Investigation, Methodology, Software, Validation, Writing - original draft, Writing - review & editing. **Vladimir Litvak:** Investigation, Methodology, Resources, Supervision, Writing - review & editing. **Esther Florin:** Conceptualization, Formal analysis, Funding acquisition, Investigation, Methodology, Project administration, Resources, Supervision, Validation, Writing - review & editing.
