# Supplementary material for: The comparative performance of DBS artefact rejection methods for MEG recordings
Source: Neuroimage. 2020 Oct 1;219:117057. doi: 10.1016/j.neuroimage.2020.117057 (PMC7443703; doi:10.1016/j.neuroimage.2020.117057)
Supplement: Multimedia component 1 [file mmc1.docx]

**Supplementary Figures**





**Supplementary Figure 1: The effects of the window size in the Hampel filter on RMSE, CTF recordings.**

6 Hz and 10 Hz window sizes lead to better results than 0.5 Hz window size.







**Supplementary Figure 2: Power spectra from 0 Hz to 140 Hz before and after applying tSSS and ICA-MI, CTF recordings.**

**Top panel:** Before artefact rejection; **Bottom panel:** After artefact rejection. Note the spectra for S3P and Hampel filter are not shown, because they were only applied to the main DBS frequency. DSMW condition shows additional peaks at lower frequencies. tSSS and ICA-MI suppress additional peaks at lower frequencies.





**Supplementary Figure 3: Power spectra from 0 Hz to 140 Hz, Elekta Neuromag recordings.**

DSMW condition did not show any additional peaks.







**Supplementary Figure 4: Individual channels at DBS freuency.**

**Top panel:** CTF recordings; **Bottom panel**: Elekta Neuromag recordings. More channels have a peak at DBS frequency for the CTF recording than for the Elekta Neuromag recording.


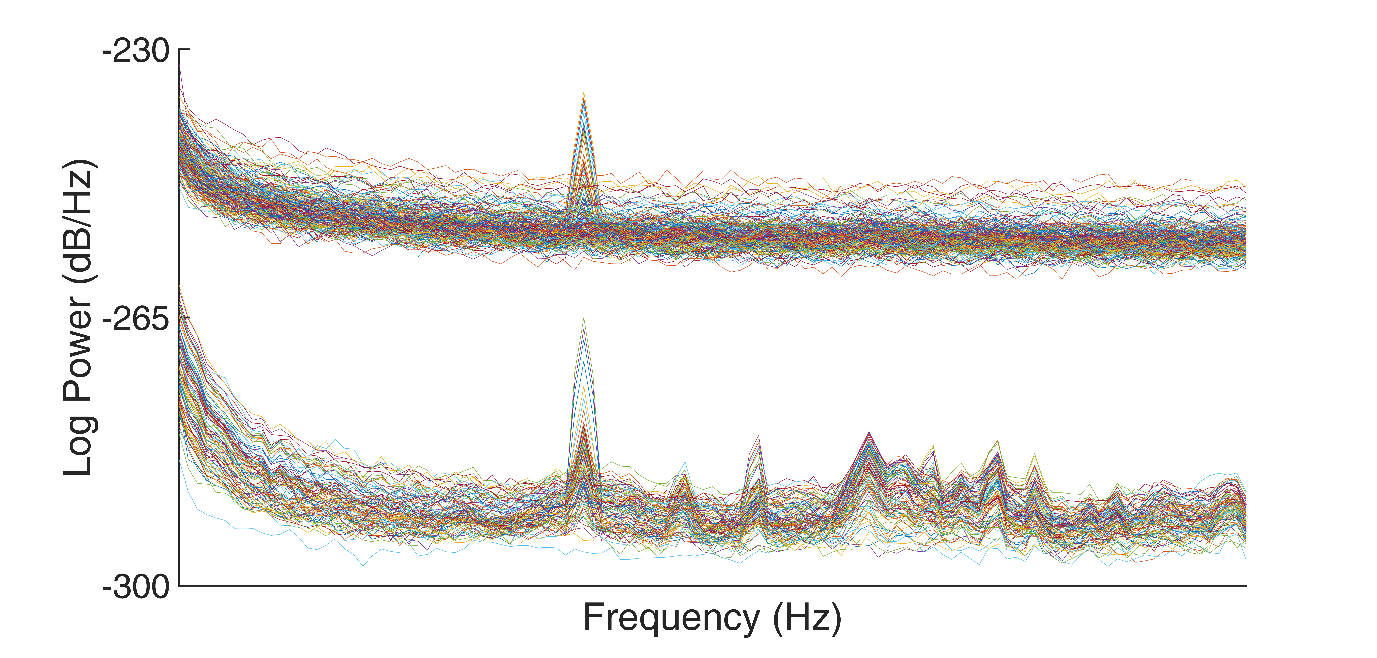





**Supplementary Figure 5: Individual channels after tSSS application on Elekta Neuromag recordings.**

**Top panel:** Reference recording in Elekta Neuromag; **Bottom panel:** Reference recording after application of tSSS CL=0.95. Each colored line represents the power spectrum of an individual channel. The amount of noise suppression differed among channels, leading to high between channel variability.
